# SARS-CoV-2 infection and transmission in school settings during the second COVID-19 wave: a cross-sectional study, Berlin, Germany, November 2020

**DOI:** 10.2807/1560-7917.ES.2021.26.34.2100184

**Published:** 2021-08-26

**Authors:** Stefanie Theuring, Marlene Thielecke, Welmoed van Loon, Franziska Hommes, Claudia Hülso, Annkathrin von der Haar, Jennifer Körner, Michael Schmidt, Falko Böhringer, Marcus A Mall, Alexander Rosen, Christof von Kalle, Valerie Kirchberger, Tobias Kurth, Joachim Seybold, Frank P Mockenhaupt, BECOSS Study Group, BECOSS Study Group, BECOSS Study Group, Esna Bozkurt, Tanja Chylla, Melanie Bothmann, Esra Demirtas, llay Gülec, Verena Haack, Franziska Haniel, Philipp Horn, Sophia Kindzierski, Mandy Kollatzsch, Marco Kurzmann, Sascha Lieber, Elisabeth Linzbach, Frederike Peters, Heike Rössig, Rafael Santos de Oliveira, Julia Steger, Zümrüt Tuncer, Vanessa Voelskow, Christof Wiesmann

**Affiliations:** 1 Institute of Tropical Medicine and International Health, Charité – Universitätsmedizin Berlin, corporate member of Freie Universität Berlin and Humboldt-Universität zu Berlin, Berlin, Germany; 2 German Red Cross Blood Transfusion Service, Frankfurt, Germany; 3 Labor Berlin - Charité Vivantes Services GmbH, Berlin, Germany; 4 Department of Pediatric Pulmonology, Immunology and Critical Care Medicine, Charité – Universitätsmedizin Berlin, corporate member of Freie Universität Berlin and Humboldt-Universität zu Berlin, Berlin, Germany; 5 Clinical Study Center, Charité – Universitätsmedizin Berlin, corporate member of Freie Universität Berlin and Humboldt-Universität zu Berlin, Berlin, Germany; 6 Medical Directorate, Charité – Universitätsmedizin Berlin, corporate member of Freie Universität Berlin and Humboldt-Universität zu Berlin, Berlin, Germany; 7 Institute of Public Health, Charité – Universitätsmedizin Berlin, corporate member of Freie Universität Berlin and Humboldt-Universität zu Berlin, Berlin, Germany; 8 The members of the BECOSS study group are listed under Investigators

**Keywords:** SARS-CoV-2, COVID-19, schools, children, adolescents, Germany

## Abstract

**Background:**

School attendance during the COVID-19 pandemic is intensely debated.

**Aim:**

In November 2020, we assessed SARS-CoV-2 infections and seroreactivity in 24 randomly selected school classes and connected households in Berlin, Germany.

**Methods:**

We collected oro-nasopharyngeal swabs and blood samples, examining SARS-CoV-2 infection and IgG antibodies by RT-PCR and ELISA. Household members self-swabbed. We assessed individual and institutional prevention measures. Classes with SARS-CoV-2 infection and connected households were retested after 1 week.

**Results:**

We examined 1,119 participants, including 177 primary and 175 secondary school students, 142 staff and 625 household members. SARS-CoV-2 infection occurred in eight classes, affecting each 1–2 individuals. Infection prevalence was 2.7% (95% confidence interval (CI): 1.2–5.0; 9/338), 1.4% (95% CI: 0.2–5.1; 2/140), and 2.3% (95% CI: 1.3–3.8; 14/611) among students, staff and household members. Six of nine infected students were asymptomatic at testing. We detected IgG antibodies in 2.0% (95%CI: 0.8–4.1; 7/347), 1.4% (95% CI: 0.2–5.0; 2/141) and 1.4% (95% CI: 0.6–2.7; 8/576). Prevalence increased with inconsistent facemask-use in school, walking to school, and case-contacts outside school. For three of nine households with infection(s), origin in school seemed possible. After 1 week, no school-related secondary infections appeared in affected classes; the attack rate in connected households was 1.1%.

**Conclusion:**

School attendance under rigorously implemented preventive measures seems reasonable. Balancing risks and benefits of school closures need to consider possible spill-over infection into households. Deeper insight is required into the infection risks due to being a schoolchild vs attending school.

## Introduction

In the coronavirus disease (COVID-19) pandemic, schooling takes a central role in the public debate. The focus is on whether schools are safe to attend, whether children, adolescents, and/or schools are relevant sources of community infections and whether school operation should be maintained, modified, or suspended [1]. Compared with adults, SARS-CoV-2 infections in children tend to take a milder or asymptomatic course, while to date, contagiousness still is debated [2,3]. However, children and adolescents temporarily had high incidences during autumn 2020 [4,5]. Modelling suggests that closure of educational facilities could notably limit overall transmission [6]. Nevertheless, there still is insufficient evidence as to whether schools actually drive the pandemic, or rather mirror it [7,8]. Observational studies on the association of school closures with community transmission have yielded inconsistent results according to a systematic review, ranging from none to substantial reduction in transmission [9]. When considering infection risks, a distinction needs to be made between school as a physical venue, students and age-typical, contextual whereabouts, e.g. public transport or after-school meetings. Limited data suggest that schools are not high-risk settings for SARS-CoV-2 transmission between students and/or staff [2,10]. On the contrary, there is evidence that school attendance itself is not a risk factor, whereas inconsistent mask use in school, contact with COVID-19 cases and gatherings outside the household are risk factors [11]. Therefore, risks need to be balanced against the detrimental impact school closures have on children and on societies’ health, social equality, workforce and economy [12,13].

Germany experienced a second pandemic wave in September 2020 and implemented a countrywide lockdown including school closures on 16 December 2020. During the peak of the second wave, we assessed SARS-CoV-2 infection and transmission in Berlin schools among schoolchildren, staff and connected household members and estimated the extent of secondary infections arising from the school context.

## Methods

### Study design, setting and participants

This was a cross-sectional analysis of a longitudinal study among students and school staff, including teachers, educators and facility staff, from 24 schools in Berlin (one class per school) and related household members. A first round of examinations of the same students and school staff had taken place in June 2020 [14]. The present second round was conducted between 2 and 16 November 2020. During that time, SARS-CoV-2 transmission in Berlin was comparatively high: 14,514 cases were recorded, and the 7-day incidence was 185–210/100,000 inhabitants [15]; the 7-day incidence in Berlin was highest in school-aged children of 10–19-year-olds (Figure). For the random selection of schools, the 12 city districts were divided into three socioeconomical strata [16]. In each stratum, two districts were randomly selected, and within these, two primary and two secondary schools. Three schools unable to participate were replaced by randomly resampled substitutes. Classes were selected among grades 3–5 (8–12 year-olds) and 9–11 (13–17 year-olds). We aimed to examine 20 students per class and up to 10 members of staff. In the first round in June, the proportion of students participating per class was 65% (range: 13–96). Hereafter, students and staff were considered index participants. In this second round in November, household members of index participants were also invited to participate.

**Figure:**
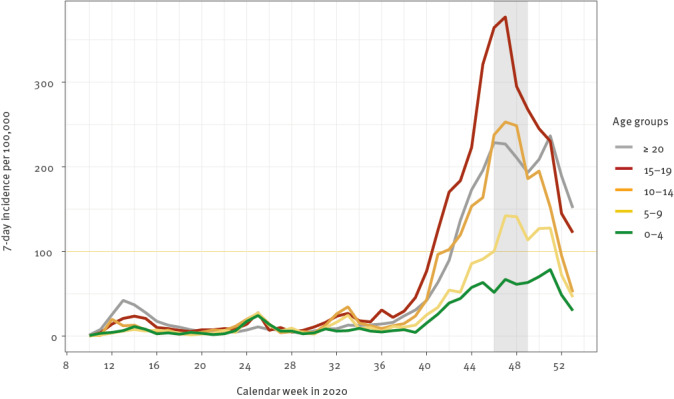
7-day incidence of recorded SARS-CoV-2 infections according to age groups in Berlin, Germany, November 2020 (n = 98,461)

SARS-CoV-2: severe acute respiratory syndrome coronavirus 2.

Study period indicated by grey shading. Data on PCR-confirmed SARS-CoV-2 infections, notified to local health authorities.

Source: Regional Office for Health and Social Affairs Berlin (LaGeSo) COVID-19 in Berlin, distribution in the districts-complete overview. https://daten.berlin.de/datensaetze/covid-19-berlin-verteilung-den-bezirken-gesamt%C3%BCbersicht (accessed: 24 October 2020).

### Data collection

Our study teams visited the schools on a scheduled day. We obtained a brief medical history from each participant and scanned forehead temperature. Fever was defined as temperature ≥ 37.5 °C. Oro-nasopharyngeal swabs (nerbe plus, Winsen/Luhe, Germany) were collected and finger-prick blood samples taken onto filter paper (BioSample Card, Ahlstrom Munksjö, Helsinki, Finland). Household members attended mobile clinics at school for symptom assessment and finger-pricking. They delivered self-collected swabs (oropharynx and nostrils), after having received instructions and swabs (CoronaOne, Berlin, Germany) beforehand. Participants absent due to illness or quarantine were visited at home, usually on the same day as the school visit. SARS-CoV-2 infection was determined by RT-PCR (GFE-Blut, Frankfurt, Germany). For anti-SARS-CoV-2-IgG, 4.75 mm dried blood spot discs were extracted in 250 µL buffer, and ELISA was performed on a EUROLabWorkstation (Euroimmun AG, Lübeck, Germany). Where we identified SARS-CoV-2 infection, health authorities were notified, participants received quarantine instructions, and over the following 14 days they were repeatedly interviewed to obtain information on health status and potential infection sources.

Participants completed a child, adolescent, or adult version of an electronic questionnaire 2 days before the study visit. Parameters assessed spanned the preceding 2 weeks if appropriate. These included household composition, signs and symptoms, contacts to SARS-CoV-2-positive persons, hand hygiene, physical distancing and wearing of facemask. Symptoms including sore throat, runny nose, nasal congestion or cough were defined as ‘cold-like symptoms’.

Lastly, we documented the school-related implementation of infection prevention and control (IPC) measures recommended by the federal state government Berlin [17]. These included hygiene measures, distancing, absence rules if ill, ventilation, cohorting, staggering of teaching hours, and online teaching. At the time of the second study round, according to governmental regulations, facemask use was obligatory when moving in hallways, but not during class. Schools decided individually if and where they implemented additional measures. We also recorded if persons were quarantined at the time of data collection. A 14-day period of mandatory quarantine was applicable for persons with exposure to an infected individual.

### Follow-up data collection

For classes where SARS-CoV-2 infection was detected, all participating students and staff and their household members self-tested again after 1 week (CoronaOne, Berlin, Germany). For systematic reasons, we retested the entire school sub-cohort (i.e, participating students and teachers), regardless of the type or amount of contact each individual of that class had with the infected person. No retesting was performed if the positive participant was already quarantined at the time of testing, i.e. did not expose classmates or staff.

### Data processing and statistical analysis

Data collection was pseudonymised. On site, data were collected on paper and subsequently entered into the Research Electronic Data Capture (REDCap) tool [18]. Descriptive analyses were segregated for primary and secondary school students, staff and household members.

We compared variables between SARS-CoV-2-infected and -uninfected participants by computing proportions, odds ratios (ORs) and 95% confidence intervals (CI). Variables of interest were socioeconomical stratum, contacts to positive cases, wearing of masks within and outside of school, hand washing, and transport to school/work. We used R version 3.6.3 for data analysis [19].

### Ethical statement

The study was reviewed by the Ethics Committee of Charité–Universitätsmedizin Berlin (EA2/091/20). We obtained informed written consent from all participants and legal representatives.

## Results

### Participants´ characteristics

We examined 1,119 participants in 24 schools including 177 primary and 175 secondary school students, 142 staff and 625 household members. Of these, 50 participants were housebound due to illness or quarantine and therefore were visited at home, or household members delivered their swabs. Seventeen students and two members of staff had dropped out or withdrawn consent since the first study round in June 2020 (not included in the 1,119 participants). The median age of primary and secondary school students was 11 and 15 years, respectively; half were female (Table 1). Staff were mostly middle-aged and female, with the majority being teachers or educators (91.2%, 114/125) in addition to facility personnel. Most household members were adults (73.8%, 461/625). Detailed symptoms are presented in Table 1. Symptoms within the preceding 2 weeks were reported by 60.2% (195/324) of all index participants, with headache (37.3%, 121/324), sore throat (15.7%, 51/324), and rhinorrhoea (14.8%, 48/324) prevailing. The most common chronic conditions reported were hypertension (2.6%, 13/494), lung disease (1.8%, 9/494), and obesity (1.0%, 5/494). Among household members, the most commonly reported symptoms over the preceding 2 weeks were headache (30.5%, 131/429), tiredness (18.6%, 80/429), and rhinorrhoea (16.8%, 72/429), while the most frequently reported chronic conditions included hypertension (4.6%, 29/624), obesity (3.7%, 23/624), and lung disease (2.1%, 13/624).

**Table 1:** Characteristics of study participants and SARS-CoV-2 symptoms, Berlin, Germany, 2–16 November 2020 (n = 1,119 participants)

Variable	Primary school students			Secondary school students				Staff				Household members		
**Number of participants**	177			175				142				625		
**Age in years (median, range), n = 1,098**	11.0 (9.0, 13.0)			15.0 (14.0, 18.0)				47.0 (28.0, 65.0)				42.0 (2.0, 86.0)		
**Sex**	Total	n	%	Total	n	%		Total	n	%		Total	n	%
Male	176	92	52.3	175	80	45.7		142	40	28.2		617	301	48.8
Female		84	47.7		95	54.3			102	71.8			316	51.2
**Reported symptoms on examination day**														
Any	177	28	15.8	174	35		20.1	141	30		21.3	600	118	19.7
Headache	177	6	3.4	174	15		8.6	141	11		7.8	600	30	5.0
Rhinorrhoea	177	19	10.7	174	14		8.0	141	9		6.4	600	62	10.3
Cough	177	6	3.4	174	5		2.9	141	7		5.0	600	36	6.0
Sore throat	177	3	1.7	174	9		5.2	141	12		8.5	600	30	5.0
Diarrhoea	177	1	0.6	174	2		1.1	141	2		1.4	600	12	2.0
Limb pain	177	2	1.1	174	0		0	141	0		0	600	8	1.3
Loss of smell or taste	177	0	0	174	0		0	141	0		0	600	9	1.5
Feeling feverish	177	3	1.7	174	1		0.6	141	1		0.7	600	7	1.2
Fever (temperature ≥ 37.5 °C), measured on-site	175	3	1.7	174	14		8.0	140	4		2.9	579	18	3.1
Any symptoms in the preceding 14 days	93	45	48.4	105	64		61.0	126	86		68.3	429	238	55.5
Any self-reported chronic condition	95	6	6.3	105	15		14.3	125	35		28.0	433	99	22.9
Regular medication	95	4	4.2	103	11		10.7	125	36		28.8	432	109	25.2
SARS-CoV-2 infection	171	6	3.5	167	3		1.8	140^a^	2		1.4	611	14	2.3
Anti-SARS-CoV-2 IgG positivity	174	2	1.1	173	5		2.9	141	2		1.4	576	8	1.4

IgG: immunoglobulin G; SARS-CoV-2: severe acute respiratory syndrome coronavirus 2.

^a^ One staff member in quarantine, not attending school.

Valid swabs were available from 347 (98.6%) students, 142 (100%) staff, and 622 (99.5%) household members; 22 specimens were lost or did not yield a result. The electronic questionnaires had a response frequency ranging from 54.9% (614/1,119) to 67.7% (758/1,119) for individual items.

### Infection prevention and control measures in schools

All schools reported the implementation of basic IPC measures such as visible signs promoting hand hygiene, providing soap and water in restrooms, and active ventilation/airing of rooms at least three times a day. Nearly half of the schools (10/22) had a hygiene commissioner. Most students (20/21 classes) and staff (22/23) reportedly adhered to hand hygiene and sneezing etiquette more than half of the time. On average, there were 26 students in each class. Three in four classes (18/24) had fixed teaching groups to prevent mixing among students, but mixing with others outside was still possible in almost all schools (22/24). Students were not supposed to attend school if they had symptoms similar to the common cold (in 19/22 classes). More than half of the classes (13/22) did not provide online teaching. In two-thirds (15/24) of the schools, wearing facemask was not obligatory in the classroom for students or staff, but outside the classroom it was obligatory for almost all schools (22/24).

### SARS-CoV-2 infections among students and staff

One or two cases of SARS-CoV-2 infection were detected in one-third (8/24) of the classes summing up to a total of 10 cases detected during the study (Table 2). These included six primary school students (two in one class, no close contact reported), three secondary students (two in one class, no close contact reported), and one secondary school staff member. The resulting prevalence in school was 2.7% (95% CI: 1.2−5.0; 9/338) among students and 0.7% among staff (95% CI: 0.0–3.9; 1/140), excluding one isolated staff member who tested positive but had already tested positive 1 week earlier. Seven of the 10 SARS-CoV-2 infected individuals were asymptomatic at testing; while two of the seven were asymptomatic throughout, the other five experienced symptoms before and/or after the test day. None of the positive index participants required hospitalisation.

**Table 2:** Characteristics of SARS-CoV-2 infections detected in schools, Berlin, Germany, 2–16 November 2020 (n = 10)

Index participant’s school type	Social stratum^a^	Ct value	Temperature ≥ 37.5 °C	Reported symptoms on test day	Symptoms before test day	New symptoms after test day	Contact with a confirmed or suspected case in preceding 2 weeks	Positive HM (n/n tested)
Primary school	High	14.1	37.6	None (but febrile at examination)	1 day before test: headache	1 day after test: loss of smell and taste	Yes, outside school	Yes (1/1)
Primary school	Low	18.9	36.1	None	6 days before test: elevated temperature, headache, fatigue for 2 days	None	None stated (other positive case in class)	No test result
Primary school	Low	17.0	36.1	Headache, cough	5 days before test: headache and fever for 2 days	None	None stated (other positive case in class)	No (0/3)
Primary school	Low	19.5	36.5	None	3 days before test: headache, eye pain	4 days after test: anosmia	Yes, at school	Yes (1/3)
Primary school	Medium	29.5	36.2	Headache, sore throat	None	None	Yes, outside of school	No (0/1)
Primary school	Low	14.7	35.2	None	None	None	None stated	Yes (3/5)^b^
Secondary school	High	21.8	37.1	None	None	7 days after test: sense of taste changed	No data	Not tested^c^
Secondary school	Medium	27.1	37.4	None	10 days before test: cold for 7 days	None	Yes, at school and other positive case in class	Not tested
Secondary school	Medium	23.3	36.6	None	7 days before test: sore throat	None	None stated (other positive case in class)	Not tested
Secondary School	Low	24.4	36.1	None	None	None	None stated	Not tested

Ct: cycle threshold; HM: household member; SARS-CoV-2: severe acute respiratory syndrome coronavirus 2.

^a^ Strata ranking is primarily characterised by indicators of unemployment, state transfer payments and corresponding income, as well as health indicators such as premature and avoidable mortality and tobacco-related serious illnesses [16].

^b^ Family members tested 4 days after student.

^c^ One family member was not tested but had tested positive elsewhere 8 days earlier.

### SARS-CoV-2 infections among household members

Fourteen members of nine households tested positive during the school-based testing, prevalence, 2.3% (95% CI: 1.3–3.8; 14/611). Nine were adults, two pre-school children, and three students at schools not participating in the study. Three family members entered the study with a 4-day delay and tested positive. Three of nine households or parts of them were in quarantine (for 3, 10, and 21 days), of which two households comprised a staff member (one of them tested positive), and in the third one, two household members were positive. From the nine positive households, six had no connection with an infected student or staff in school, whereas three did. For the three positive households with a positive student in school, extensive review could not establish the origin of infection.

Half (7/14) of the household members infected with SARS-CoV-2 reported cold-like symptoms on the test day. Among the asymptomatic individuals, most reported symptoms before or after the test date; one adult was briefly hospitalised (Table 3).

**Table 3:** Characteristics of SARS-CoV-2 infections among household members, detected during the school survey in Berlin, Germany, 2–16 November 2020 (n = 14)

Index participant’s school type; household number	Positive index household member in school	Social stratum^a^	Ct value	Temperature (≥ 37.5 °C); self-measured	Reported present symptoms	Symptoms before test day	New symptoms after test day	Contact with a confirmed or suspected case in preceding 2 weeks	Total number of positive household members at testing (n/n tested)
Primary school; H1	Yes	High	19.3	37.9	None (but febrile at examination)	None	1 day after test: fever, cough, felt very ill for 14 days	Yes	2/2
Primary school; H2	No	High	20.4	36.0	None	5 days before test: feverish	5 days after test: limb pain, weakness, felt very ill for 14 days	No	1/3
Primary school; H3	No	Low	25.9	37.2	Cough	None	None	None stated	2/4
Primary school; H3	No	Low	25.2	36.7	Sore throat	None	None	None stated	2/4
Primary school; H4	Yes	Low	26.1	36.4	None	3 days before test: cold symptoms	Anosmia	None stated	2/4
Primary school; H5^b^	No	Low	23.5	36.3	Rhinorrhoea, cough	3 days before test: cold symptoms for 10 days	None	Yes	1/4
Primary school; H6	Yes	Low	11.9	NA	None	5 days before test: limb pain, anosmia	2 days after test: fever, feeling very ill, after 7 days hospitalised for 5 days receiving oxygen	None stated	4/6
Primary school; H6	Yes	Low	21.9	NA	None	14 days before test: cough	None	None stated	4/6
Primary school; H6	Yes	Low	22.5	NA	None	14 days before test: mild cold	None	Yes	4/6
Secondary school; H7^b^	No^b^	Low	21.6	36.3	Cough	14 days before test: fever and cough for 3 days	None	Yes	3/3
Secondary school; H7^b^	No^b^	Low	19.4	36.3	Rhinorrhoea	10 days before test: start of cold	None	Yes	3/3
Primary school; H8^b^	No	Low	21.4	NA	Rhinorrhoea, anosmia	None	None	Yes	2/5
Primary school; H8^b^	No	Low	16.9	NA	Rhinorrhoea, cough, anosmia	Cold symptoms	None	Yes	2/5
Secondary school; H9	No	Low	24.8	35.7	None	14 days before: mild cold	None	No data	1/5

Ct: cycle threshold; H: household; NA: not available; SARS-CoV-2: severe acute respiratory syndrome coronavirus 2.

^a^ Strata ranking is primarily characterised by indicators of unemployment, state transfer payments and corresponding income, as well as health indicators such as premature and avoidable mortality and tobacco-related serious illnesses [16].

^b^ In quarantine.

Age not reported to avoid identification of participants.

### SARS-CoV-2 IgG antibodies

Anti-SARS-CoV-2 IgG antibodies were present in 2.0% (95% CI: 0.8–4.1; 7/347) of students, 1.4% (95% CI: 0.2–5.0; 2/141) of staff, and 1.4% (95% CI: 0.6–2.7%; 8/576) of household members. Among infected participants, two of 21 showed anti-SARS-CoV-2-IgG, and three of 21 had borderline reactivity. Five presently uninfected index participants who had no antibodies in June 2020 did so in the present study. None was aware of previous infection. Thus, 1.1% (95% CI: 0.4–2.6; 5/449) of students and staff had been infected with SARS-CoV-2 without noticing.

### New SARS-CoV-2 infections at follow-up after 1 week

Students, staff, and household members connected to eight classes with SARS-CoV-2-positive index participants were retested after 1 week. Students and staff of five of the eight affected classes had been quarantined within a median of 3 days (range: 1–5) after the initial test day. In three schools, only close contacts were quarantined. Among 381 tested individuals (who had tested negative or not tested at baseline), seven (1.8%) new infections were detected at retesting. Of note, no school-related infection of students or staff was observed at retesting. Although two index participants tested positive at follow-up (Table 4), we classified their infections as not school-related. In the first case, a secondary school student was retested because of a positive member of staff, but direct contact was excluded. Instead, the student’s household member tested positive and developed symptoms a few days before the student. In the second case, a staff member had been at home to take care of a positive household member, and was tested positive at follow-up. Furthermore, five household members (four adults, one child) tested positive at follow-up. Except for the mentioned household member of the positive index student, the remaining four had a positive child in school 1 week before. For two of them, we assumed SARS-CoV-2 transmission via a positive index participant, and for two household members, this remained unclear. Consequently, we conservatively estimated the attack rate following 10 infections in eight school classes as 1.1% (95% CI: 0.3–2.9; 4/352 persons with exposed index participant at cross-sectional assessment).

**Table 4:** Characteristics of SARS-CoV-2 cases at follow-up testing after 7 days, Berlin, Germany, 9–25 November 2020 (n = 7)

Index participant’s school type	Index participant or HM	In index participants: case in class?	Linked index participant positive at testing?	Other HM positive?	Infection assumed to be school-related?^a^	Social stratum^b^	Ct value	Temperature (≥ 37.5 °C)	Present symptoms during testing	Symptoms after testing	Cumulative number of HM tested positive (n/n tested)^c^
Primary school	HM	NA	Yes	Yes	Unclear	High	15.8	NA	Headache, limb pain, anosmia, cough	Cough for approx. 14 days	3/4
Primary school	HM	NA	Yes	NA	Unclear	Low	21.0	36.4	None	None	2/2
Primary school	Index participant	No	NA	Yes	No	Low	22.5	36.9	Headache, feeling feverish	Headache, 1 day after test: anosmia, 3 days after: weakness	2/4
Primary school	HM	NA	Yes	Yes	Yes	Low	13.6	36.4	Limb pain, dizziness	2 days after test: anosmia, dizziness, weakness, cough for 1 month	3/4
Secondary school	HM	NA	Yes	NA	Yes	Medium	24.4	36.7	None	None	2/4
Secondary school	Index participant	No^d^	NA	No	No	Low	21.8	36.5	Headache, diarrhoea	1 day after: cold, limb pain, diarrhoea, dizziness	2/3
Secondary school	HM	NA	No	No	No	Low	10.7	36.2	Cold symptoms	Strong cold symptoms for a total of 5 days (started 2 days before test)	2/3

Ct: cycle threshold; HM: household member; NA: not applicable; SARS-CoV-2: severe acute respiratory syndrome coronavirus 2.

^a^ Based on review.

^b^ Strata ranking is primarily characterised by indicators of unemployment, state transfer payments and corresponding income, as well as health indicators such as premature and avoidable mortality and tobacco-related serious illnesses [16].

^c^ Including those from cross-sectional testing.

^d^ No fixed class in this school subcohort; positive staff identified in school at cross-sectional testing; but no contact between those two cases, therefore not regarded as a secondary case.

Age not reported to avoid identifiability of participants.

As to manifestation, two positive individuals were asymptomatic at retesting, whereas the others reported mainly cold-like symptoms (Table 4).

### Comparison of SARS-CoV-2 infected and non-infected participants

SARS-CoV-2 infection was present in 4.7%, 1.9%, and 1.0% of classes located in the low, medium, and high socioeconomical strata, respectively (high vs low; OR = 4.71; 95% CI: 0.82–48.18; Table 5). Almost nine in 10 index participants stated to wear a facemask often or always at school, and their infection prevalence was 1.4%. Of those who wore masks never to sometimes, 14.3% tested positive (OR = 11.38; 95% CI: 2.28**−**59.64). Similarly, eight of the 16 non-affected classes and one of the eight affected classes reported a facemask obligation in classroom. While contact to a suspected or confirmed COVID-19 case in school did not confer increased odds of infection, such contacts outside school tended to do so (infection prevalence: 8.3%; OR = 3.52; 95% CI: 0.56**−**16.27). Lastly, infection tended to be more common in those who reported to walk to school (without other means of transport; prevalence, 8.2%; OR = 3.84; 95% CI: 0.76**−**16.82).

**Table 5:** Comparison between SARS-CoV-2 negative and positive index participants, Berlin, Germany, 2–16 November 2020 (n = 478)

Variables	Negative, n = 467		Positive, n = 11		OR	95% CI
	n	%	n	%		
**Sex**						
Female	269	97.8	6	2.2	1	Ref
Male	197	97.5	5	2.5	1.14	0.27–4.54
**Socioeconomical stratum^a^**						
High	193	99.0	2	1.0	1	Ref
Medium	151	98.1	3	1.9	1.92	0.22–23.18
Low	123	95.3	6	4.7	4.71	0.82–48.18
**Contact to suspected or confirmed case at school**						
No	213	96.4	8	3.6	1	Ref
Yes	92	97.9	2	2.1	0.58	0.06–2.98
**Contact to suspected or confirmed case outside of school**						
No	271	97.5	7	2.5	1	Ref
Yes	33	91.7	3	8.3	3.52	0.56–16.27
**Mask wearing frequency at school**						
Often to always	273	98.6	4	1.4	1	Ref
Never to sometimes	30	85.7	5	14.3	11.38	2.28–59.64
**Mask wearing frequency in public**						
Often to always	299	97.4	8	2.6	1	Ref
Never to sometimes	5	100	0	0.0	NA	NA
**Hand washing frequency**						
0–1 times	6	85.7	1	14.3	1	Ref
2–4 times	92	98.9	1	1.1	0.07	0.00–5.97
≥ 5 times	206	96.7	7	3.3	0.20	0.02–10.70
**Transport to/from school/work ** **Exclusively by foot**						
No	259	97.7	6	2.3	1	Ref
Yes	45	91.8	4	8.2	3.84	0.76–16.83
**Exclusively by bicycle**						
No	227	96.6	8	3.4	1	Ref
Yes	77	97.5	2	2.5	0.74	0.07–3.81
**Exclusively by car**						
No	249	96.1	10	3.9	1	Ref
Yes	55	100	0	0.0	NA	NA
**By public transport (exclusively, or in combination with other means of transport)**						
No	206	97.2	6	2.8	1	Ref
Yes	98	96.1	4	3.9	1.40	0.28–6.06

CI: confidence interval; NA: not applicable; OR: odds ratio; Ref: reference; SARS-CoV-2: severe acute respiratory syndrome coronavirus 2.

^a^ Strata ranking is primarily characterised by indicators of unemployment, state transfer payments and corresponding income, as well as health indicators such as premature and avoidable mortality and tobacco-related serious illnesses [16].

Among household members, infection was more prevalent in the low compared with the high socioeconomical stratum (OR = 12.37; 95% CI: 2.68–114.84), and in those who had contact to a suspected or confirmed case outside of work or school (OR = 5.76; 95% CI: 1.37–21.96; data not shown).

## Discussion

Our results from schools during peaking SARS-CoV-2 transmission in November 2020 in Berlin are: in one third of the classes, one or two infections were detected, mostly asymptomatic. Connected household members in 2.3% were also infected; a school-related origin of infection was unlikely in two third of cases. No secondary infections occurred in the affected classes within 1 week. The attack rate in households connected to positive classes was 1.1%. Infection prevalence in school was increased in case of rare wearing of facemask in school, walking to school, low socioeconomical stratum, and case-contacts beyond school.

The SARS-CoV-2 prevalence of 2.7% among students in our study exceeds results of similar studies in Germany and other highly affected European countries in that period. Among more than 2,500 students and staff in Saxony, Germany, in November 2020, 1.0% were infected with SARS-CoV-2; seroprevalence was 1.4% [20]. A concurrent study in Austrian students reported 0.4% SARS-CoV-2 prevalence [21], while in more than 100 English schools, 1.2% of students and 1.3% of staff were infected [5]. During data collection, the 7-day incidence in Berlin among those aged 15–19-years exceeded that of younger ages. This accords with higher infection figures in secondary than in primary school students [4], but contrasts our findings. We cannot exclude an incidental finding; differences in hygiene and distancing might also be involved [22], e.g., mask wearing was not mandatory at primary schools. Only one of 140 attending school staff was infected at cross-sectional testing. This is in line with data from England, where SARS-CoV-2 infection was present in 0.4% of teachers, similar to other professions, arguing against increased infection risks among school staff [5]. More than half of all participants reported mainly cold-like symptoms in the preceding 2 weeks, and about one in five on the test day. During study conduct, acute respiratory infections in Germany occurred at less than half the rate of previous years, probably due to enhanced hygiene measures [23]. However, surveyed symptoms are subjective, and health consciousness might increase during a pandemic, possibly causing overestimations. Yet, seven of 10 positively tested index participants were asymptomatic and would thus not have been identified by symptom-based testing. Similarly, five index participants had unknowingly developed antibodies. This stresses the benefit of routine testing in schools, which is now widely recommended and implemented [24].

When comparing SARS-CoV-2-uninfected and -infected index participants, the latter tended to attend school in the low socioeconomical stratum. School stratum was a rough proxy disregarding intra-district variability of education, occupation and income, but other research concurringly showed that social disadvantage and SARS-CoV-2 infection in students is associated [21]. Moreover, household infection clusters in our study occurred largely at low socioeconomical stratum. This may reflect household crowding with insufficient distancing and isolation possibilities promoting transmission. Increased infection prevalence was observed among those who inconsistently used facemasks in school. Wearing a facemask in school was not obligatory at that time, but many schools and classes nevertheless adhered to it. Prevalence was similar among participants with and without case-contacts in school, but with case-contacts outside of school, infection tended to be more prevalent. This corroborates findings from Mississippi, United States, where attending lessons was not found to be a risk factor for SARS-CoV-2 infection among students, but inconsistent mask wearing in school, close case-contacts outside the household, and social gatherings [11]. Lastly, prevalence was increased among those who walked to school. Lacking conclusive evidence, we suspect that grouping up with friends on the way to school was a possible reason.

In the connected households, SARS-CoV-2 prevalence of 2.3% was observed. Only for three of nine affected households, a school-link was assumed. At retesting, no school-related secondary infection was seen among students and staff of eight affected classes, despite ongoing exposure before quarantine. For the connected households, the attack rate was 1.1%. This suggests that, even at high epidemic activity, attending lessons in school is not a major place of transmission if adequate IPC measures are implemented. So far, only few larger school outbreaks have occurred in Germany [25,26]. In the federal state of Rhineland-Palatinate between August and December 2020, school surveillance yielded a secondary attack rate among primary contacts of around 1% [27]. A simultaneous investigation in neighbouring Hesse found an average secondary attack rate of 1.3% among contact persons in school [28]. Similarly, in Italy, there was a low prevalence in schools and low intra-school transmission in the study timeframe up to October 2020 [29].

These findings of a rather low level of transmission in the school context are difficult to reconcile with results indicating substantial effects of school closures. In observational data from the United States from early 2020, school closure was associated with significant declines in COVID-19 incidence and mortality [30], whereas in a systematic review of observational studies, effects of school closure are inconsistent [9]. Several modelling studies - usually from the first wave of the pandemic - suggest modest to substantial associations between school closures and incidence [6,31]. These include estimates of 40–60% reduced peak incidence [31], and of reducing the reproduction number by more than a third [6]. Moreover, school closures have been associated with an overall mobility reduction of 21.6% in Switzerland [32]. It remains difficult to disentangle the direct or indirect consequences of school closure from those of other non-pharmaceutical interventions, which were frequently implemented in parallel [30]. For example, school closures imply less mobility, but also substantial disruptions in daily routines, particularly for parents, and altered working conditions, childcare, and social contacts. Recent evidence shows that incidence in school and population are linked [5]. Similarly, our data suggest that most detected infections were not acquired in school. In class, students experience clear guidelines regarding preventive behaviour and respective enforcement. Such rules, e.g. wearing of facemasks and airing, may partially explain the rather low infection figures despite grouping in class. In contrast, during school closures, students possibly assemble in uncontrolled settings [33]. Conceivably, shutting down educational facilities brings about transmission reductions which are not directly attributable to attending classes and intra-school transmission, but to indirect consequences including parental behaviour. If that were true, pandemic mitigation measures would need to focus more strongly on indirect patterns, e.g. mandatory filtering masks in public, offering more frequent public transport to avoid overcrowding, and obligatory work from home wherever possible. However, there is a lack of information to delineate the respective impact on the COVID-19 pandemic during school closures. This is all the more regrettable when considering the many harmful consequences of this measure for children and society [12,13].

The strengths of our study are random selection of schools across Berlin, school-based generation of empirical data rather than model-based estimates, inclusion of connected households, solid laboratory methods, and screening rather than symptom-based testing allowing for the detection of asymptomatic infections. The limitations of our study are a low number of outcome events and potential selection bias (voluntary participation). Comparative data on the prevalence of SARS-CoV-2 in the Berlin underage population are not available. Incomplete swabbing due to self-administration cannot completely be excluded despite illustrated instructions and PCR quality control including human RNase P gene co-amplification.

## Conclusion

SARS-CoV-2 infection activity in Berlin schools during peak transmission in November 2020 appeared to be low. Secondary transmission in class was absent, and in connected households, the attack rate was around 1%. Based on our findings, we are cautiously optimistic that schooling itself does not necessarily lead to child-to-child transmission or constitutes a central COVID-19 pandemic driver, provided that IPC measures are rigorously implemented. Our study is longitudinal and the continuation of our study will show whether this is true as the determinants of the pandemic change, including vaccination coverage, population immunity, relaxed or tightened lockdown, and viral mutations. Our findings do not exclude the possibility of school-based outbreaks, particularly at higher transmission or enhanced viral transmissibility. Repeat screening in schools to detect asymptomatic infections is justified by our data and should help reducing the infection burden [34]. As a prerequisite for further, tailored measures, deeper insight is needed into the fraction of infections attributable to being a school child as compared to school attendance itself.
